# Copper and Copper Proteins in Parkinson's Disease

**DOI:** 10.1155/2014/147251

**Published:** 2014-01-08

**Authors:** Sergio Montes, Susana Rivera-Mancia, Araceli Diaz-Ruiz, Luis Tristan-Lopez, Camilo Rios

**Affiliations:** Neurochemistry Department, National Institute of Neurology and Neurosurgery “Dr. Manuel Velasco Suárez”, Insurgentes Sur 3877, Colonia La Fama, 14269 Tlalpan, DF, Mexico

## Abstract

Copper is a transition metal that has been linked to pathological and beneficial effects in neurodegenerative diseases. In Parkinson's disease, free copper is related to increased oxidative stress, alpha-synuclein oligomerization, and Lewy body formation. Decreased copper along with increased iron has been found in *substantia nigra* and caudate nucleus of Parkinson's disease patients. Copper influences iron content in the brain through ferroxidase ceruloplasmin activity; therefore decreased protein-bound copper in brain may enhance iron accumulation and the associated oxidative stress. The function of other copper-binding proteins such as Cu/Zn-SOD and metallothioneins is also beneficial to prevent neurodegeneration. Copper may regulate neurotransmission since it is released after neuronal stimulus and the metal is able to modulate the function of NMDA and GABA A receptors. Some of the proteins involved in copper transport are the transporters CTR1, ATP7A, and ATP7B and the chaperone ATOX1. There is limited information about the role of those biomolecules in the pathophysiology of Parkinson's disease; for instance, it is known that CTR1 is decreased in *substantia nigra pars compacta* in Parkinson's disease and that a mutation in ATP7B could be associated with Parkinson's disease. Regarding copper-related therapies, copper supplementation can represent a plausible alternative, while copper chelation may even aggravate the pathology.

## 1. Introduction

Parkinson's disease is an age-associated chronic condition; it is the second most common neurodegenerative disorder, affecting an important fraction of world population. It is estimated that in 2040, in the US alone, the population aged 65 years and older will be as high as 80 million [1]. The costs, at the personal and national health care system levels, continue to rise [1]. The mean age at the onset of Parkinson's disease is 55 years [2], and its prevalence dramatically increases after this age.

Clinically, Parkinson's disease is characterized by four cardinal symptoms: tremor at rest, muscle rigidity, slowness of movement (bradykinesia, akinesia), and changes in posture (instability). Usually, tremor begins unilaterally and then becomes bilateral [3]. Motor symptoms develop distal, and thus, although tremors in the hands are frequently the first observed, tremors in the face are also common. Walking can be especially difficult for patients; because of the postural instability, patients have a tendency to fall. As a whole, this combination of symptoms leads to disability and dependency. Parkinson's disease patients, in the long term, become dependent on others for daily living activities, such as dressing or feeding, and, therefore, the quality of life of patients suffering from Parkinson's disease is considerably diminished [4]. Along with movement alterations, Parkinson's disease patients show very important and debilitating nonmotor symptoms, such as autonomic dysfunction, cognitive abnormalities, sleep disorders, mood disorders, pain, and sensory disorders [4, 5].

The main pathological hallmark of Parkinson's disease is the loss of dopamine-producing neurons, whose cell bodies are located in the *substantia nigra pars compacta,* as well as the presence of aggregates of misfolded proteins (mainly alpha-synuclein) and other materials, known as Lewy bodies [6]. The dopaminergic cells in this region project terminals to the caudate/putamen nuclei; therefore, as a consequence of cell death, a decreased dopamine content is observed in the basal ganglia, leading to the motor symptoms observed in patients. Although a considerable portion of the cases of this disease are linked to genetic defects [7], the causes behind Parkinson's disease are uncertain in the vast majority of cases, and the disorder is considered multifactorial. In this regard, many theories have been developed to explain the cause of protein aggregation and mechanisms underlying cell loss, including the overproduction of free radicals associated with mitochondrial dysfunction (either cause or consequence), alterations of the ubiquitin-proteasome system, inflammation, and exposure to environmental pollutants [8–10]. Alterations in transition metal storage, transport, and cellular handling have gained attention in neurodegenerative diseases [11], particularly as early *postmortem* reports showed iron accumulation and decreased copper levels in brains from Parkinson's disease patients [12, 13]. This finding is especially important because both metals are involved in the generation and propagation of free radicals, as well as in protein precipitation, as a result of their redox properties. Copper is a special case because, in addition to the previously mentioned mechanism of damage, it is also a cofactor of antioxidant enzymes such as Cu/Zn-SOD and ceruloplasmin. This duality makes copper interesting for the study of neurodegenerative diseases. The objective of the present review is to gather and summarize information concerning copper, copper-related proteins, and mechanisms of damage and protection related to Parkinson's disease pathophysiology.

## 2. Free Copper as a Cause of Damage 

There is important evidence in the literature concerning the role of free copper as deleterious for neurodegenerative diseases; this review focuses on its role in Parkinson's disease. The main effects of copper are mediated by its redox capacity and thus by its ability to initiate, maintain, or even potentiate the generation of free radicals. In addition, copper has been involved in the inclusions of proteins (as consequence of misfolding or fibrillation) and gains of function in copper enzymes. An issue that must be highlighted is that, in most cases, the damaging aspects of copper are present when this metal is present as free ion or linked to low molecular weight ligands, is released from copper-containing enzymes due to the surrounding conditions, or shows evident alterations of transport/storage.

Occupational studies have noted that long-term exposure (20 years) to copper and manganese increases the risk of Parkinson's disease [14]. In agreement with these studies, other environmentally based studies within urban populations have shown that the incidence of Parkinson's disease is greater in those areas with important emissions of copper or manganese; in such cases, the relative risk for copper was 1.1, within a 95% confidence interval from 0.94 to 1.31, meaning that copper exposure barely reached significance [15].

A meta-analysis performed by Mariani and cols. [16] considering copper and iron levels in the serum, plasma, and CSF showed no differences between Parkinson's disease patients and controls; they found that meta-analysis contributed to an increased dispersion of data analyzed and, thus, that the differences observed in individual studies were diluted. Therefore, they performed a replication study with newly recruited Parkinson's disease patients and controls, but again, they found no differences from the results reported for other Parkinson's disease populations [17].

Copper is a transition metal that, similar to iron, participates in the cascade of free radical generation as a catalyst in Fenton chemistry [18], which also involves hydrogen peroxide (this is especially important in brain areas metabolizing biological amines such as dopamine because hydrogen peroxide is a byproduct of the monoamine oxidase metabolism). The Fenton reaction, Cu(I) + H_2_O_2_↔ Cu(II) + OH^−^ + OH^∙^, turns the relatively stable hydrogen peroxide into the highly reactive hydroxyl radical, which is known to react with lipids, proteins, and nucleic acids [18]. It is important to note that in order for copper to act as a catalyst for the Fenton reaction, at least two conditions must be fulfilled: (1) the oxidation state for copper must be +1 and (2) the ion should be free. The second may be debatable, but it is more probable to occur in this way because the free ion is more diffusible and available. In this regard, some groups have reported that copper concentrations in CSF are higher in Parkinson's disease patients than in controls [19–21] and, furthermore, that free copper in CSF can be related to clinical variables, even being used as biochemical marker of the disease [20]. The increased free copper in CSF could imply that copper is “leaking” from proteins or cells or that it is not adequately transported in or out of cells, as will be discussed later. Free copper in CFS may also have other interpretations, considering that tissue from Parkinson's patients is diminished in areas intimately related to the pathology such as the caudate nucleus or the *substantia nigra* [12, 22]. Therefore, one may consider that the loss of copper in those areas could be an important event, not only for the release of ions that may contribute to more free radical generation but also because they are no longer available for the maintenance of endogenous antioxidant systems, that is, CU/Zn-SOD. In addition, copper is related to iron control metabolism. As will be discussed later, decreased copper may be linked to iron accumulation through a decreased ferroxidase activity, effected mainly by ceruloplasmin [23]; the activity of this enzyme has been continuously reported as diminished in samples from patients with Parkinson's disease [20, 24, 25]. Iron accumulation in the basal ganglia represents a problem by itself because it is well known that iron participates in the formation and propagation of reactive oxygen species [26]. In fact, the direct injection of iron into the *substantia nigra* causes tissue damage and decreased dopamine and metabolites, which is considered a model of Parkinson's disease [27].

In an interesting *in vitro* study, Spencer et al. [28] showed that copper ions facilitate the oxidation of dopamine and other related catechols, such as L-Dopa and 6OH-dopamine. The complexes resulting from dopamine oxidation products and copper were observed to cause intense damage to DNA. The findings in this paper are relevant because they suggest another mechanism of damage to copper, specifically in the dopamine-rich areas implicated in Parkinson's disease. Although the effect of copper relies on an oxidative mechanism, this effect differs from the catalysis of copper in the Fenton chemistry mentioned above.

Some studies dealing with the copper load in the organism have been carried out; from these, a recent study showed that rats exposed to copper (1 mg/L) in the drinking water for four weeks presented not only liver damage, measured as increased serum transaminases, but also an increase of 50% in the brain metal content. The overload of copper was linked to oxidative parameters such as diminished GSH and lowered SOD activity, as well as to increased levels of the lipid oxidation marker malondialdehyde [29].

The direct injection of copper sulfate into the *substantia nigra* of rodents has also been tested, and the authors assayed several copper doses, finding that noxious copper effects began at 50 nmoles intranigral, which is fivefold greater than the values of iron necessary to produce damage. This injury consisted of decreased dopamine, increased oxidative stress, and apoptosis with a sensitive loss of TH immunoreactivity [30]. In this paradigm, copper served as a toxin for dopaminergic cells, mainly because it was injected as a free ion and thus acted as a catalyst for free radical overproduction.

Lewy bodies are intracellular inclusions formed mainly by alpha-synuclein, a protein of unknown function that is present close to synaptic terminals; the oligomerization of this protein is considered a key event in the setup or development of the disease [31]. One of the reported copper-damaging mechanisms is the oligomerization of alpha-synuclein [32]; in fact, it is claimed by some authors that copper is highly efficient in producing the oligomerization of alpha-synuclein [33] and that this metal, and not iron, is selectively able to fibrillate alpha-synuclein [34]. That was also related to the ability of copper to cause oxidative damage because the alpha-synuclein oligomerization is linked to damage to the mitochondria and electron chain transfer.

## 3. Copper as an Essential Metal

Apart from the information described in the preceding paragraphs, copper is necessary for cellular physiology, and copper is considered an essential metal [35]. There is a complex system for the absorption, distribution, storage, and handling of this transition metal; the collection of mechanisms of transport will be considered in a proper section ahead in this review. In this section, we discuss the general physiological roles of copper, while the relationship of specific copper proteins and Parkinson's disease will be discussed in the following section.

The mutation of genes involved with copper transport shows two extreme cases regarding brain copper, deficiency and overload. In the case of deficiency, the mutation of the ATP7A transporter causes Menkes disease, which is characterized by severe deficiency of copper all over the organism. Individuals carrying the defective gene die at an early age, evidencing the pivotal role of copper in development. Menkes patients show severe seizures and a disruption of the brain energy metabolism [36]. On the other extreme, Wilson's disease results from the mutation of a different transporter protein, ATP7B, which is involved in the transport of copper into the bile and ultimately in copper excretion [37]. Individuals suffering from Wilson's disease show excess of brain copper, basal ganglia degeneration, movement disorders, psychiatric manifestations, and cirrhosis because of the copper burden in the body [38]. Again, the clinical manifestations of both diseases indicate the importance of maintaining appropriate copper levels.

Copper is required in a myriad of reactions in cell metabolism [39], particularly in the brain because this organ has a high respiratory rate and is prone to oxidative stress. In this regard, one of the most important physiological functions of copper-dependent proteins relies on their redox capacity. Copper not only participates in the quenching of reactive oxygen species as a cofactor in Cu/Zn-SOD but also contributes to the electron transport chain in cytochrome C, which transfers electrons between Complexes III (Coenzyme Q-Cyt C reductase) and IV (Cyt C oxidase) at the inner membrane of mitochondria. It also participates in neurotransmitter synthesis (dopamine beta-hydroxylase), neurotransmitter metabolism (diamine oxidase and monoamine oxidase), the handling or storage of other metals (metallothioneins, ceruloplasmin, and haephestin), and extracellular matrix formation (lysyl oxidase), among other functions [39]. Some other proteins related to central nervous system pathologies have recently gained attention because they show an affinity for copper. It is now recognized that the interaction between copper and proteins could be a key event for neurodegenerative diseases.

The participation of copper in the brain physiology is not limited to its incorporation into redox-sensitive proteins; for example, Schlief and cols. [40] showed that in cultured hippocampal neurons, copper is released by the neural stimulation of NMDA-type glutamate receptors. The release of copper was observed as a process that required a calcium signal but did not operate by the classic fusion of neurotransmitter vesicles with the membrane. In addition, NMDA receptor activation induced the copper transporter ATP7A to relocate into synaptic active zones. This effect is related to the replenishment of synapsis-related copper, indicating that the continuous use of the glutamatergic synapsis ensures the availability of copper to be released. Other studies have shown that copper is enriched in synaptic vesicles [41], with even synaptosomes being able to reuptake copper. The copper concentration on the synaptic cleft ranges from 0.2 to 1.7 *μ*M, but the intracellular neuronal concentrations can reach up to 3-fold that value. This indicates that specific systems are activated to concentrate copper inside the cell and to keep it for release upon stimulation, rendering hundred micromolar concentrations in the synaptic space during neuronal activity. As a whole, this evidence suggests that copper plays a messenger, signaling role in the synaptic space.

Electrophysiological studies have shown that, in low concentrations, copper is able to inhibit currents induced by NMDA agonists [42], suggesting allosteric modulation in excitatory glutamate signals. The studies of Vlachová et al. [43] showed that copper occupies a different site in the NMDA than that of glutamate or glycine. The IC50 for copper inhibition in the patch clamp preparation was 0.27 *μ*M, implying some potency in the modulation of NMDA receptor. AMPA/Kainate receptors in the cortex are also modified by copper (IC50 = 4.5 *μ*M) [44]. Other studies have characterized the effect of copper in the hippocampus; it was found that copper at a concentration of 50 *μ*M was able to disrupt long-term potentiation by a mechanism involving presynaptic AMPA receptors [45]. The effects of copper are complex and time-dependent. While the acute exposure of the neurons to copper produced a blockage of neurotransmission, when cells were preexposed to the metal 3 hours before recording, copper facilitated the glutamatergic response in an AMPA receptor-mediated manner [46]. This finding was related to the recruitment of new receptors in the membrane and the anchoring of the PSD95 protein. The authors further concluded that the release of copper in the glutamatergic synapse seems to enhance and maintain communication between cells [46].

Copper has also shown modulatory properties in GABA A receptors from cortex membranes, due to the reduction of Cl^−^ currents in a concentration-dependent fashion [47]. Further studies have determined that copper inhibits currents in GABA receptors with an IC50 = 2.4 *μ*M [48]. Copper seems to act in the gating system of the GABA receptor channel [36]. A recent study has shown copper blocks with far more potency against extrasynaptic GABA receptors than synaptic GABA A receptors. In such conditions, copper may interfere with the tonic inhibition elicited by GABA [49].

Electrophysiological studies in neurons from rat olfactory bulbs have revealed some other actions of copper at the synapsis, such as the inhibition of TTX-sensitive sodium channels, delayed responses in rectifying K^+^ channels, and the inhibition of voltage-dependent calcium channels [50].

## 4. Copper-Binding Proteins in Parkinson's Disease

Several copper-dependent enzymes and copper-binding proteins are known to exist [23]. Here, we review some of them, but we restrict our review to those involved in Parkinson's disease through either protective or damaging mechanisms.

### 4.1. Alpha-Synuclein

Alpha-synuclein is a protein of unknown function that is enriched at the presynaptic terminals of many neurons. Alpha-synuclein is strongly implicated in Parkinson's disease and other neurodegenerative disorders, such as dementia with Lewy bodies, multiple system atrophy, and Alzheimer's disease (nucleopathies). All of these diseases are characterized by intracellular aggregations of proteins called Lewy bodies, which are particularly rich in filamentous alpha-synuclein [51].

Alpha-synuclein is present in the plasma and cerebrospinal fluid of healthy subjects and Parkinson's patients [52, 53]. An important issue to highlight is that oligomer protein levels are higher in Parkinson's individuals than in paired subjects; therefore, the polymerization of alpha-synuclein, although not exclusive to the disease, is clearly related. It has been observed that the duplication or triplication of the gene encoding alpha-synuclein is related to a familiar form of the disease [54]. Alpha-synuclein is physiologically catabolized in the cell by the ubiquitin-proteasome system, which is defective in patients with idiopathic forms of the disease [55]. Alpha-synuclein aggregation causes dysfunction of the ubiquitin-proteasome system [56]. Based on this evidence, cell loading with alpha-synuclein seems to be another factor that could influence fibrillation and the formation of intracellular inclusions.

The alpha-synuclein protein binds copper. Although some studies have suggested different quantities of metal per copy of the protein, the most consistent results show two sites for copper binding per monomer at nanomolar and micromolar concentrations [57]. These sites implicate a histidine residue in position 50 and the carboxy terminal fragment as important sites for metal binding. Thus, alpha-synuclein has high affinity for copper and is very effective at causing its fibrillation, a phenomenon that yields further precipitation of similar proteins and is ultimately thought to represent the “seeding” that gives rise to Lewy bodies [32]. This is in accordance with the fact that copper is encountered in Lewy bodies at relatively high concentrations.

The binding of copper to alpha-synuclein is an important event for the setup/development of the disease because several interrelated consequences seem to derive from it. The first, as has already been discussed, is the conformational change of alpha-synuclein that facilitates fibrillation and aggregation [32, 33]. There is experimental evidence showing that the copper-alpha-synuclein complex induces changes in copper's redox properties, and, thus, this complex has been linked to increased H_2_O_2_ production from ascorbic acid oxidation and, in turn, the dopamine oxidation by H_2_O_2_. The Cu-alpha-synuclein complex itself is also capable of oxidizing other endogenous antioxidants, for example, GSH [58]. An interesting study by Davies and cols. [59] showed that recombinant alpha-synuclein binds both copper and iron; the loading of copper into the protein produced small changes in the iron binding kinetics, suggesting different binding sites for both metals. Furthermore, alpha-synuclein showed ferrireductase activity. These authors linked their findings with a physiological need for Fe(II) in dopamine synthesis through tyrosine hydroxylase and with a pathological scenario, involving the participation of Fe(II) in the Fenton reaction, increasing the oxidative stress of Parkinson's disease.

### 4.2. Ceruloplasmin

Ceruloplasmin is a multicopper containing glycoprotein that is mainly biosynthesized in the liver [60]. Copper-bound ceruloplasmin is released to the blood by the liver, where the newly formed enzyme is bound to copper early in its synthesis in the secretory pathway, along with the incorporation of a polysaccharide moiety [61]. Ceruloplasmin acts as an iron oxidase, copper transporter, amine oxidase, antioxidant, anti-inflammatory, nitric oxide oxidase, and glutathione peroxidase, among other actions [62]. The most prominent functions of ceruloplasmin are the following: (a) plasma copper binding (95% of circulating copper is bound to ceruloplasmin) and (b) iron homeostasis by means of its ferroxidase activity, with implications for the control of free radical production, as discussed below.

The soluble form of ceruloplasmin in the blood is involved in the oxidation of the iron to be incorporated into transferrin [62]; therefore, severe copper deficiency is characterized by diminished ferroxidase activity, leading to iron retention in the liver and defective iron distribution to the other organs.

In the brain, ceruloplasmin is synthesized by astrocytes, where it is anchored to the plasmatic membrane and linked to glycosylphosphatidylinositol [63]. The glial-derived ceruloplasmin is intimately linked to iron efflux from the brain because, as in the liver, ceruloplasmin oxidizes iron that has been previously transported by ferroportin to be incorporated into transferrin [26]. Aceruloplasminemia, a genetic condition producing a lack of function of circulating ceruloplasmin, is characterized by iron accumulation, remarkably in the brain, where it is associated with neurodegeneration and extrapyramidal parkinsonian symptoms [64]. In fact, diseases known to involve the accumulation of iron in the brain, for example, aceruloplasminemia, ferritinopathy, and a syndrome of neurodegeneration with brain iron accumulation, are characterized by neuronal death and motor manifestations similar to those of Parkinson's disease [26].

As it has been continuously mentioned, the basal ganglia of Parkinson's disease patients show iron accumulation and decreased copper [12, 13]; those findings could be in agreement with the fact that copper-dependent ferroxidase activity promotes the oxidation of Fe^2+^ to Fe^3+^ [24] so that Fe^3+^ can be removed from the brain. Therefore, it is possible that the decreased content of copper in the *substantia nigra* is related to the increased iron in this area, as a result of the defective ferroxidase activity. Accordingly, copper-dependent ferroxidase activity has been reported to be diminished in the plasma and cerebrospinal fluid of patients with Parkinson's disease [17, 20, 24]. Iron accumulation seems to be an important feature in the setup or development of Parkinson's disease; for example, it has been found that iron, observed by magnetic resonance imaging, begins to accumulate in the *substantia nigra* before the appearance of parkinsonian symptoms. It has also been proposed that iron signals in the *substantia nigra* can be a predictive marker of the disease [65]. Other studies have reached a similar conclusion; taking advantage of iron echogenicity and of the temporal bone window at the mesencephalon level in humans, it is possible to determine echogenic areas in the *substantia nigra* by using transcranial ultrasound [66]. Further studies with postmortem tissues from Parkinson's disease patients have shown that increased echogenicity is related to iron [67]. The specific accumulation of iron in the s*ubstantia nigra* has served to propose the echogenicity of this region as a characteristic feature of Parkinson's disease or even a prognostic index of the disease's development. In fact, a negative correlation between brain iron accumulation and copper-dependent ferroxidase plasma activity [68] has been reported. Other studies have confirmed this phenomenon; using magnetic resonance imaging, two populations of Parkinson's disease patients were found: those with increased brain iron and those with no apparent iron accumulation. It is worth noting that the first group also showed diminished ferroxidase activity [69].

In a study determining serum ceruloplasmin (protein, not the ferroxidase activity), a positive correlation between ceruloplasmin and the age of onset of Parkinson's disease was found; the stratification of the sample with a cut-off point of 60 years as the age of onset showed decreased ceruloplasmin in the serum in earlier onset patients. Serum copper was not different between the early and late onset Parkinson's disease groups; however, both groups showed decreased copper levels in comparison with a control group [70].

A mechanism of ceruloplasmin dysfunction regarding its ferroxidase activity was proposed in the study by Olivieri et al. [71]; they found that the electrophoretic pattern of ceruloplasmin was different between CSF samples from Parkinson's disease patients and controls and that changes were related to oxidative modifications, for example, protein carbonylation. The experimental oxidation of ceruloplasmin produced a similar pattern to those obtained from patient's CSF. Furthermore, oxidized ceruloplasmin produced an accumulation of iron in cultured cells, whereas functional ceruloplasmin prevented the iron load and stimulated the synthesis of proteins related to iron storage and efflux. The authors further discussed the implications of oxidized ceruloplasmin because this event would lead not only to alterations in the iron efflux from the brain but also to the possibility of releasing copper from the enzyme, yielding free copper ions to be available for the production of even more free radicals.

Further evidence of ceruloplasmin malfunction in Parkinson's disease has also been derived from studying the ceruloplasmin gene; five variants of ceruloplasmin were found in a screening study in a cohort of patients who displayed increased *substantia nigra* echogenicity [72].

In a recent study, Ayton et al. [25] found that postmortem *substantia nigra* from Parkinson's disease patients showed increased iron and decreased copper; no differences were observed in the ceruloplasmin protein levels. However, they did find severely reduced ferroxidase activity. They also found that deficient ceruloplasmin mice displayed neuronal cell death in the *substantia nigra* and that neurodegeneration was partially reduced by the use of an iron chelator. Finally, they found that the peripheral administration of ceruloplasmin (5 mg/kg, i.p.) partially prevented both the increased iron in the *substantia nigra* and the dopaminergic cell death induced by MPTP. Ceruloplasmin, due to its antioxidant properties and its role as an iron regulator in the brain, remains an attractive target for new therapeutic strategies in Parkinson's disease.

### 4.3. Cu/Zn-SOD

Superoxide dismutases are a group of enzymes that catalyze the reaction of superoxide to hydrogen peroxide [73]. The function carried out by those enzymes in the brain is important because superoxide derives from multiple sources in the cell metabolism, such as the electron transport chain as a product of one electron oxygen reduction, and NADPH oxidase. Particularly for dopaminergic areas, the metabolism of dopamine by monoamine oxidase has superoxide anion as a byproduct [74]. The cytoplasmic (type I) and extracellular (type III) superoxide dismutase isoforms require copper and zinc as cofactors, whereas the mitochondrial type II isoform is Mn-dependent [75].

It is remarkable to note that, in a wide variety of studies (either with human tissues or in experimental animals), the overexpression of superoxide dismutase has been constantly found to be neuroprotective; this fact underscores the importance of oxidative stress in Parkinson's disease and the importance of SOD by itself.

Experimental evidence shows that the overexpression of Cu/Zn-SOD in mice provides them with resistance to the dopaminergic neurotoxin MPTP, with regard to dopamine depletion [76]. In a study by Nakao et al. [77], *substantia nigra* from embryonic mice overexpressing human Cu/Zn-SOD and from wild type controls were grafted onto the nigra of immunosuppressed rats. The rats were then injured with the 6-OHDA toxin to model Parkinson's disease. Grafts derived from transgenic mice overexpressing SOD showed a 4-fold increase in the survival of TH cells compared to those from littermates with a regular expression of the enzyme. Cells not only survived better but also showed normal function.

Microglial cells transfected with human Cu/Zn SOD1, when properly stimulated, are able to release the extracellular isoform of superoxide dismutase into the medium; this antioxidant enzyme in turn protects neurons against 6-OH dopamine-induced cell damage [78]. Accordingly, the exposure of cultured astrocytes to dopamine favored the selective expression of extracellular Cu/Zn-SOD (not SOD 1 or Mn SOD), depending on the dopamine concentration itself (not receptors or metabolism) and nuclear factor kappa-B. The authors suggest that astrocytes may be able to protect the surrounding cells by expressing extracellular SOD [79]. The protein DJ-1, which is the product of the familiar gene for Parkinson's disease, PARK7, enhances the expression of type I Cu/Zn-SOD in connection with the Erk1/2-Elk1 cascade. DJ-1 null mice were more susceptible to the injection of the toxin MPTP due to the failure of SOD upregulation [80].

Physical exercise has been reported as a protective factor in Parkinson's disease and other neurodegenerative diseases suspected to coincide with oxidative stress [81]. In this regard, experimental studies suggest that physical activity induces SOD and other antioxidant enzymes [82].

Ihara et al. [83] found that blood from Parkinson's disease patients showed an increased concentration of hydroxyl radicals and diminished Cu/Zn-SOD in red blood cells. Parkinson's disease patients with a higher concentration of hydroxyl radicals in the plasma were related to an earlier onset of the disease and higher Hoehn and Yahr stages. A previous report showed no differences in the SOD activity between Parkinson's disease patients and age-matched controls; however, in Parkinson's patients, the SOD activity decreased significantly with the duration of the disease [17], suggesting faster deterioration of the antioxidant ability of Cu/Zn-SOD in Parkinson's disease. Studies carried out with postmortem tissues have confirmed that aging reduces the capacity of antioxidant enzymes, including SOD, in *substantia nigra* only selectively, suggesting that this region is especially susceptible, as indicated by the progression of Parkinson's disease [84].

### 4.4. Other Copper-Binding Proteins

Metallothioneins are a family of low molecular weight proteins composed of a high number of cysteine residues, conferring them with the ability to bind metals [85]. In the brain, metallothioneins I and II are expressed in the glia, whereas metallothionein III is expressed in neurons and metallothionein IV is expressed in different epithelia [86]. Physiologically, metallothioneins are linked to zinc homeostasis in the brain. The high affinity of metallothionein for copper and the described release of copper in glutamatergic synapsis suggest that metallothioneins may buffer copper ions in the vicinity of the synapsis. In fact, metallothionein III binds zinc and copper under physiological conditions [86]. Metallothioneins (I and II) can be upregulated in different situations, such as metal intoxication, steroid use, and oxidative stress. In most of the cases, metallothioneins exert a neuroprotective role [87, 88]. The study of metallothioneins in Parkinson's disease is appealing because these enzymes are able not only to bind free metal ions but also to scavenge directly for free radicals. Studies carried out with postmortem tissues from patients with Parkinson's disease showed increased expression of glial metallothionein I in the *substantia nigra* and cortex; the authors considered that the observed effect could be a compensatory mechanism to protect glial cells from oxidative stress [89]. It has been observed in rodents that MPTP-induced extrapyramidal damage reduces the expression of metallothionein I [90] and other antioxidant enzymes. Parkinson's disease may affect the endogenous antioxidant systems as a mechanism for disease development. The transgenic mice overexpressing metallothionein I were shown to be more resistant to the peroxynitrite-releasing agent SIN-1; these animals were also more resistant to the MPTP model or Parkinson's disease than nontransgenic controls. The effect herein observed was related to increased coenzyme Q10 synthesis [91].

As already mentioned, the binding of alpha-synuclein to copper results not only in protein fibrillation but also in an increased oxidative stress. In this regard, Meloni and Vašák [92] found that the complex alpha-synuclein-Cu(II) is able to oxidize dopamine to o-quinone in the presence of oxygen; the process involved the cycling of Cu(II) to Cu(I) and back. By changing dopamine with ascorbate, the authors found that the hydroxyl radical was produced. The incubation of these complexes with metallothionein III showed that the copper ion originally bound to alpha-synuclein was transferred to metallothionein, at which point the oxidative effects, dopamine oxidation, and production of hydroxyl radicals were abolished. Because metallothionein III is expressed in neurons, regulating this protein seems a promising strategy, at least in experimental Parkinson's disease paradigms.

Amyloid precursor protein possesses a copper-binding domain that possesses copper reductase activity [93]. Amyloid precursor protein knockout mice exhibit high copper levels in the cortex and liver. These findings were the basis of a suggestion that APP is a membrane copper transporter [93]. In turn, animals overexpressing APP show decreased copper in the brain [94]. Additionally, there are reports providing evidence of the ability of APP to enable ferroxidase activity. It is considered that APP could be fulfilling the action of oxidizing iron and exporting it from cells by an association with ferroportin in neurons, similar to the mechanism of the reported effect of ceruloplasmin in astrocytes. Amyloid precursor protein knockout mice fed with a high iron diet displayed increased iron levels both in the brain and the liver; this effect was in turn related to oxidative stress alterations, such as reduced glutathione and increased protein carbonylation. In the same study, the cortical ferroxidase activity assigned to APP was reduced only in samples from Alzheimer's disease and not from Parkinson's disease [95]; thus, the ferroxidase activity of APP in the basal ganglia in Parkinson's disease remains to be fully elucidated.

A recent study noted that the DJ-1 protein is able to bind copper and mercury [96]. DJ-1 expression causes the cells to become more resistant to either copper or mercury because DJ-1 confers protection against copper-induced oxidative stress. However, this protection is lost when oxidized dopamine is present in the medium. This finding supports other studies showing that mutant rodents lacking DJ-1 are more susceptible to MPTP [97].

In addition to their function of editing amyloid precursor proteins, presenilins are related to copper transport and homeostasis. In fact, it is claimed that presenilin ablation may diminish the activity of copper-dependent SODs because of the decreased availability of intracellular copper [98]. Further studies from the same group confirm the role of presenilins in the transport of copper and the consequences of limited copper for the synthesis of copper-dependent antioxidant enzymes [99].

The fact that prion proteins may accept a considerable number of copper ions into the extracellular space of the synapsis has served to suggest an interesting theory regarding prion proteins as a buffering control for copper at the synapsis [100]. The binding of copper to prion proteins confers its SOD activity [101], giving rise to the hypothesis of the gain of function for prion proteins as a key event in the misfolding of the proteins and the propagation of these altered forms.

## 5. Copper Transport

For the reasons expressed above, maintaining adequate copper levels is essential to preserving normal brain functions. For example, copper concentration in the cerebrospinal fluid is up to 100-fold lower than values in plasma [102] when the cytosolic concentration of unbound copper is very low [103].

Under physiological conditions, most plasma copper ions are bound to ceruloplasmin, with a small proportion of copper being carried by albumin, transcuprein, and other amino acids [104]. However, according to experimental evidence, neither copper-albumin nor copper-ceruloplasmin uptake represents a significant contribution to copper transport, compared to free copper uptake into brain capillaries, the choroid plexus, and CSF [105, 106]. Copper enters the brain mainly via the BBB (blood-brain barrier), although the BCB (blood-cerebrospinal fluid barrier) is also able to transport it into the brain to a much lesser extent [102, 107]. It is believed that epithelial cells from the choroid plexus serve as a reservoir for copper [108].

At the BBB, CTR1 (copper transporter-1), ATP7A, and ATOX1 (antioxidant 1 copper chaperone) are all involved in copper transport into the brain [109]. The blood-CSF barrier seems to maintain copper at a certain level by sequestering copper from the blood and exporting the excess out of the CNS and back to the blood [109], as demonstrated by Monnot et al. [107], who found that the main direction for copper transport is from the apical to the basolateral side of epithelial cells. At the BCB, copper transport is regulated by two major copper transporters: CTR1 and divalent metal transporter-1 (DMT1) [110]. These transporters, together with ATP7A, transport copper from CSF to the blood, while ATP7B together with CTR1 achieves this in the opposite direction [109]. The significance of the participation of DMT-1 in brain copper transport is still controversial, but it is known that, in the epithelial cells of the choroid plexus, there is a coupling between copper and iron homeostasis that involves this transporter [109].

### 5.1. Copper Transporter 1 (CTR1)

CTR1 is a plasma membrane protein having three transmembrane domains that form a homotrimeric pore for copper uptake [111]. CTR1 is present in the brain capillary endothelial cells of the BBB, choroid plexus of the BCB, and the brain parenchyma [105, 112, 113].

CTR1 is responsible for transporting copper in the intestinal cells and is profusely expressed in brain capillary endothelial cells, where it is considered the major pathway for copper transport from the blood into the brain [114]. This transporter is also expressed abundantly in the choroid plexus and, as opposed to its function in the BBB, transports copper out of the brain in the BCB [109].

CTR1 is concentrated on the apical surface in cells of the choroid plexus; it is also found in the cytoplasm of neurons in the visual cortex, anterior cingulate cortex, caudate, and putamen and in cytoplasm of Bergmann glia in human tissue. It is distributed around neuromelanin granules in the *substantia nigra* [115], most likely regulating the acquisition of copper by this pigment [116].

Studies from Davies et al. [115] suggest that, in the normal human brain with adequate cellular copper, CTR1 exists primarily as an internalized protein pool, rather than as an active membrane-bound transporter, whereas at high copper levels, it is internalized into the cell and subsequently degraded [117].

To our knowledge, there are no reports about any mutations of CTR1 in Parkinson's disease; only a marked reduction of neuronal CTR1 immunoreactivity and correlation between CTR1 and copper levels in the *substantia nigra* of Parkinson's disease postmortem human brains have been described [118]. This reduction in CTR1 levels can be very important because neural tissue is particularly sensitive to the loss of CTR1 function, as indicated by marked cell death in the brain and spinal cord of zebrafish in response to CTR1 downregulation [119]. This occurrence could be attributable to the copper depletion caused by a decreased level of its main known transporter to allow it to enter the brain. More studies are needed to establish the relevance of CTR1 in Parkinson's disease.

### 5.2. Antioxidant 1 Copper Chaperone (ATOX1)

Human ATOX1 is a small cytosolic protein of 68 amino acids [114]. In solution, ATOX1 exists as a monomer, but, in the presence of metals, it can form dimers [120]. ATOX1 is expressed abundantly in the pyramidal neurons of cerebral cortex, hippocampus, and *locus coeruleus*; moderately in the olfactory bulb; and little in the cerebellum (except for Purkinje neurons) [121, 122].

The copper chaperone ATOX1 is involved in the delivery of copper to ATP7A and ATP7B inside the cells [109, 114, 123]. ATOX1 levels correlate positively with copper content in the human brain [115] and function as an antioxidant against superoxide and hydrogen peroxide [124].

ATOX1-mediated copper transfer is accompanied by the upregulation of the Cu-ATPase's activity, while apo-ATOX1 can retrieve copper from the ATPases and downregulate their activity [125].

Changes in the ATOX1 levels appear to induce remodeling of the entire copper-metabolic network [114]. Cultured Atox1−/− cells exhibit increased ATP7A levels, whereas Atox1−/− newborn mice show low activity of several copper-dependent enzymes [123, 126, 127].

As far as we know, mutations or changes in the ATOX1 levels in Parkinson's disease have not been described. This is an interesting molecule to study because of the functions described above.

### 5.3. P-Type ATPases ATP7A and ATP7B

ATP7A and ATP7B are members of the P1B-subfamily of the P-type ATPases; they catalyze the translocation of copper across cellular membranes by ATP-dependent cycles of phosphorylation and dephosphorylation [102]. They have eight transmembrane domains that form a path through cell membranes for copper translocation and a large N-terminus with six metal-binding domains (MBDs), each comprising approximately 70 amino acids and the highly conserved metal-binding motif GMxCxxC (where x is any amino acid) [102].

ATP7A is expressed in the brain capillaries, choroid plexus, astrocytes, and neurons from mice [105, 128–130]. In both the epithelial cells of the choroid plexus and the capillary endothelial cells of the brain, ATP7A is predominantly located on the basolateral membrane, while ATP7B concentrates at the apical membrane [102].

ATP7A and ATP7B are expressed in neuronal cell bodies in some brain regions, and both of them are expressed in the cytoplasm of neurons in the *substantia nigra*; only ATP7B is associated with neuromelanin [115].

ATP7A and ATP7B are able to deliver copper for incorporation into copper-dependent enzymes and to remove excess of copper from cells, depending on their subcellular location [102]. ATP7A is important in the delivery of copper from endothelial cells to the brain [130], which has been confirmed by the fact that mice with a mutated ATP7A gene accumulate copper in brain capillaries and suffer from copper deficiency in the brain.

ATP7B, as opposed to ATP7A, is expressed in brain capillaries more than in the choroid plexus [105] and it is possibly involved in copper transport from the blood to the CSF [109].

ATP7A and ATP7B levels are not associated with copper brain levels, but their cellular location changes as copper levels are modified [115]. At physiological conditions, ATP7A and ATP7B are mainly located in the trans-Golgi network to incorporate copper into cuproenzymes; when copper levels are high, these proteins are redistributed to post-Golgi vesicles and even to the cellular membrane to facilitate copper export [109, 131, 132].

Because ATP7A has faster kinetics of copper transport in relation to ATP7B, a predominant homeostatic role for ATP7A and a biosynthetic role for ATP7B have been proposed as mediators of the synthesis of cuproenzymes [133]. Enzymes such as cytochrome c oxidase, SOD1, DBH (dopamine *β*-hydroxylase), PAM (peptidylglycine *α*-amidating monooxygenase), lysyl oxidase, and tyrosinase require ATP7A for metallation in the trans-Golgi network [102].

The known information about these ATPases comes mostly from their study in Menkes disease and Wilson's disease. Menkes disease is caused by a mutation in the gene encoding the copper transporter ATP7A that results in severe copper deficiency in the brain [134], and Wilson's disease is caused by a mutation in the gene encoding the copper transporter ATP7B, resulting in copper accumulation in the brain [135]. A specific role for these two transporters in Parkinson's disease has not been thoroughly investigated, but there is at least one study that relates ATP7B to Parkinson's disease to some extent [136].

While Wilson's disease is an autosomal recessive disorder caused by mutations in the ATP7B gene [137], it has been hypothesized that a single mutated ATP7B allele may act as a risk factor for (late-onset) parkinsonism [136, 138]. Sechi et al. [136] found a nucleotide deletion at the 5′UTR region in a single allele of ATP7B gene in three sisters with levodopa-responding parkinsonism; mutations in other Parkinson's disease-related genes were not found in any of the sisters.

On the other hand, as Parkinson's disease is an aging-related disease, we consider that it is important to understand the behavior of molecules that have some relationship with its pathophysiology. In this respect, Lenartowicz et al. [139] studied ATP7A expression in the mouse liver from P.05 to P240. They found that the expression of ATP7A decreases during the lifespan; in fact, the ATP7A expression in adult mice is very low in comparison with that in neonatal and young animals. Interestingly, the same behavior was observed for liver copper levels [139]. It would be interesting to study the behavior of ATP7A expression in the brain as a function of age and its implications on copper homeostasis.

ATP7B supplies copper to cuproenzymes such as ceruloplasmin [140]. As discussed previously, ceruloplasmin activity is decreased in Parkinson's disease; to our knowledge, there are no studies that show or refute any relationship between ATP7B dysfunction and decreased ceruloplasmin activity in Parkinson's disease.

### 5.4. DMT1

DMT1, also known as divalent cation transporter 1 (DCT1), transports one proton and one atom of Fe(II) in the same direction, and it also performs a nonselective transport for multiple divalent metals, including Mn, Cu, Co, Zn, Cd, and Pb [141, 142]. While the presence of DMT1 in the BBB remains controversial, there are data supporting its presence in the BCB [110, 142, 143], although the experiments of Zheng et al. [110] suggest a minimum contribution of DMT-1 in cellular copper uptake in the BCB.

The upregulation of DMT1 in the *substantia nigra* of Parkinson's disease patients and in the *substantia nigra* of mice exposed to 1-methyl-4-phenyl-1,2,3,6-tetrahydropyridine (MPTP), a neurotoxin known to induce several features of Parkinson's disease, has been demonstrated [144]. Additionally, the CC haplotype derived from single nucleotide polymorphisms (SNPs) of DMT1 was found to be a possible risk factor for Parkinson's disease in the Han Chinese population [145]. These alterations of DMT1 in Parkinson's disease are believed to affect iron transport significantly but not copper transport.

## 6. Copper-Related Therapies

The current therapeutic strategies, such as supplying a dopamine precursor (L-DOPA), dopamine agonists (e.g., pramipexole, bromocriptine), and inhibitors of dopamine breakdown (e.g., selegiline), similar to surgical ablations or deep brain stimulation, only provide symptomatic relief of the motor impairment [146]. There is still an imperative need to move from symptom-alleviating to disease-modifying therapies [147].

As discussed before, the role of copper in Parkinson's disease is controversial, as some evidence points to a need for increased copper levels, while other results show the opposite. There have been some attempts made to clarify the roles of the two pathways, which will be discussed below.

Regarding the possibility of increasing brain copper levels, two main options can be tested as follows: (1) to regulate copper transporters to increase copper entry into the brain or (2) to administer copper compounds (or copper-releasing compounds). Regarding the first option, further knowledge of the function of copper transporters in Parkinson's disease is needed; regarding the second alternative, some strategies have been already tested.

Using the rodent model of Parkinson's disease induced by MPP^+^ (1-methyl-4-phenylpyridinium) intrastriatal injection, our group found that the administration of CuSO_4_ (10 *μ*mol/kg i.p.) as a pretreatment 24 h before the lesion prevented protein nitration, TH inactivation, and dopamine depletion and decreased the activity of constitutive nitric oxide synthase (cNOS) in the striatum [148]. It is possible that copper antagonizes NMDA receptor responses by inhibiting Ca^2+^ influx and thus inhibiting Ca^2+^-dependent NOS activation, reducing protein nitration [148]. Recently, using the same paradigm, we found that copper pretreatment increased ceruloplasmin expression and prevented the MPP^+^-induced loss of ceruloplasmin ferroxidase activity and the concomitant increase in lipid peroxidation. Additionally, a slight decrease in ferrous iron was found in the striatum and mesencephalon [149]. We consider that the increased ferroxidase activity is responsible for the decline in ferrous iron content and the concomitant prevention of lipid peroxidation. As such, copper-induced ceruloplasmin expression could be an experimental strategy against the deleterious effects of iron deposits in Parkinson's disease.

The use of the hypoxia imaging agent Cu(II) (atsm) in four different models of Parkinson's disease has been shown to be neuroprotective by several mechanisms, including the inhibition of alpha-synuclein nitration and fibrillation. The copper compound also showed the protection of dopamine-producing neurons by TH immunostaining as well as the preservation of motor function and reduced cognitive decay [146].

EGb761 (an extract of the *Ginkgo biloba* tree) pretreatment also blocks the neurotoxic actions of MPP^+^ [150]; some of the protective actions of this extract can be attributed to the reversing of the MPP^+^-induced copper depletion in the striatum of rats and the regulation of copper homeostasis in other brain regions [151].

Taking into account the possibility of increased copper levels in Parkinson's disease, it has been suggested that copper chelation can be useful in the treatment of some neurodegenerative diseases, including Parkinson's disease [21, 152]. However, copper chelation was not protective against MPTP injury [153] and even, as in the case of diethyldithiocarbamate, enhanced neurotoxicity [154]. It is known that iron is very harmful in Parkinson's disease and that copper reduces Fe uptake, possibly through DMT1 [155].

There are some studies suggesting that Fe accumulation is a consequence of copper deficiency. Increased ferroportin expression is associated with neuronal survival after Fe overload [155]. Copper-deficient diets reduce ferroportin expression in the rat liver [156], possibly leading to Fe accumulation; in patients with nonalcoholic fatty liver disease, a low hepatic copper content is associated with a decreased ferroportin expression, thus contributing to Fe accumulation [156]. In rats fed a copper-enriched diet, the influx of Fe into the brain was significantly decreased compared to that of rats fed with the control diet [157]. According to those studies, Fe accumulation may be the consequence of copper deficiency. Supporting this hypothesis, Fe accumulates in several tissues during copper deficiency [155].

On the other hand, in a study of patients with smell dysfunction, Henkin et al. [158] reported that, after repetitive transcranial magnetic stimulation (rTMS), patients had increased copper concentrations in the plasma, erythrocytes, and saliva, showing that rTMS can produce changes in copper homeostasis. rTMS has been used with some success to treat the clinical manifestations of patients with Parkinson's disease, resulting in improved motor performance, elevation of serum dopamine, and improved smell and taste functions [158–161]. It is not known whether changes in the copper levels at a systemic level are a reflex of changes in brain levels of copper or whether the improvement observed in patients with Parkinson's disease and other neurological disorders after rTMS is due, at least in part, to modifications in the copper levels.

The experimental evidence discussed here shows that a deficiency of copper in Parkinson's disease is more possible than an excess and that copper supplementation can be a plausible alternative to treating Parkinson's disease. However, due to the delicate equilibrium in copper homeostasis and the need for research about the distribution of copper in different compartments of the brain and other organs, therapeutic strategies trying to adjust the copper levels in the brain must be undertaken with caution. The severe consequences of copper deficiency and overload can be illustrated by Menkes disease and Wilson's disease, respectively. Additionally, although copper is required for the oxidation of Fe^2+^ to avoid oxidative damage, too much copper is also toxic.

## 7. Conclusions

Among the interesting facts regarding copper-binding proteins, we found that alpha-synuclein bound to copper acts as a ferrireductase, thus increasing the availability of iron for the generation of free radicals; this could be particularly important in the caudate/putamen vulnerable regions of the brain, because of the presence of the oxidative labile dopamine. In addition, copper-dependent ferroxidase activity of ceruloplasmin has been continuously reported to be reduced in samples from Parkinson's disease patients. Theoretically, the decreased function of ceruloplasmin would aggravate the abovementioned situation of alpha-synuclein. Accumulation of brain iron is an event unambiguously related to the disease, but the proportion in which copper or copper proteins are responsible for iron dyshomeostasis in Parkinson's disease is not known accurately. The role of copper transporters in Parkinson's disease is an issue that also deserves further research. Knowledge about copper compartmentalization in brain will help to establish promissory therapeutic strategies aimed at enhancing the positive role of this metal in Parkinson's disease.
